# Beyond Body Mass Index: The Impact of Height and Height‐Normalised Weight on Overall Survival of Lung Cancer Undergoing Surgery

**DOI:** 10.1002/jcsm.70049

**Published:** 2025-08-18

**Authors:** Lorenzo Gherzi, Mathilde Prieto, Antonio Iannelli, Laurent Brouchet, Pierre‐Benoit Pagès, Pierre Emmanuel Falcoz, Françoise Le Pimpec Barthes, Pascal Alexandre Thomas, Marcel Dahan, Marco Alifano

**Affiliations:** ^1^ Thoracic Surgery Department Cochin Hospital, University of Paris Paris France; ^2^ University of Nice Côte d'zur, Nice, France; ELSAN, Clinique Saint Michel, Centre de Chirurgie de l'Obésité (CCO), Toulon, France; Adipocible Research Study Group University of Nice Côte d'Azur and Initiative d'Excellence ‐ Idex Nice France; ^3^ Thoracic Surgery Department Hôpital Larrey, CHU Toulouse Toulouse France; ^4^ Thoracic Surgery Department CHU Dijon, 1 BD Maréchal de Lattre de Tassigny Dijon France; ^5^ Thoracic Surgery Department, Nouvel Hôpital Civil de Strasbourg University of Strasbourg Strasbourg France; ^6^ Thoracic Surgery Department Hôpital Européen Georges Pompidou, University of Paris Paris France; ^7^ Thoracic Surgery Department Hopital‐Nord‐APHM, Aix‐Marseille University, Chemin des Bourrely Marseille France

**Keywords:** BMI, height, lung cancer

## Abstract

**Introduction:**

Unlike most malignancies, increased adiposity, as expressed by a higher body mass index (BMI), is associated with improved prognosis after lung cancer surgery at the population level. Height, one of the determinants of BMI, is associated with better survival, independent of other confounders, even though BMI is calculated as weight divided by height squared. The association of weight with survival is difficult to assess because, at the individual level, weight is closely linked to height and does not directly reflect adiposity. In this study, we examined the impact of height and weight on overall survival in a large population of patients undergoing upfront surgery for lung cancer.

**Methods:**

We extracted data on all consecutive patients with stage I–IIIA non‐small cell lung cancer included in a surgical nationwide dataset over a 16‐year period. For each sex, height was categorised in sex‐specific quartiles (sH). Sex‐specific height‐normalised weight (sHNW) was defined as the ratio of an individual's weight to the mean weight of individuals of the same sex and height, and it was categorised into quartiles. Finally, the sum of the category membership (ranging from 1 to 4 according to quartiles) of sH and sHNW was calculated, and the results were categorised into four groups of sH/sHNW. Overall survival (OS) was assessed by Kaplan–Meier, and differences evaluated by log‐rank. Cox models were built.

**Results:**

The study included 50 653 patients. Mean age was 65.61 ± 9.45 and 31.99% were women. sH predicted OS, taller height being protective [crude HRs of second, third, and fourth quartiles vs. first quartile: 0.94 (95% CI 0.91–0.98), 0.89 (0.86–0.92), 0.77 (0.74–0.81); *p* < 0.0001]. sHNW was also associated with OS, with lower sHNW category being associated with worse outcome and higher sHNW categories being protective [crude HRs of second, third and fourth quartiles vs. first quartile: 0.88 (0.85–0.92), 0.82 (0.79–0.85), 0.85 (0.81–0.88); *p* < 0.0001]. The four classes of sH/sHNW showed higher differences in prognosis with respective crude HRs of 0.88 (0.84–0.93), 0.76 (0.73–0.80) and 0.70 (0.66–0.74) in the intermediate lower, intermediate higher and higher class as compared with the lower class. Five‐year overall survival rates were 58.65% (56.89–60.45), 62.96% (62.15–63.78), 67.71% (67.02–68.41) and 70.12% (68.98–71.26), in the lower, intermediate lower, intermediate higher and higher class, respectively. All Cox models showed that sHNW and sH/sHNW predicted survival independently from common confounders.

**Conclusions:**

Our study demonstrated that sHNW and sH/sHNW are strong prognostic factors of resectable lung cancer. This finding could have both epidemiologic and biological relevance.

## 1 Introduction

Disease stage is currently the most important prognostic factor in non‐small‐cell lung cancer (NSCLC) and remains a cornerstone in determining optimal treatment [1, 2, 3]. In recent years, host‐related factors such as systemic inflammation, nutritional status or sarcopenia have been found to influence long‐term survival in resectable disease, independent of tumour stage [4, 5, 6, 7, 8]. Lower body mass index (BMI) at diagnosis has been shown to be associated with decreased long‐term survival after resection of NSCLC compared with normal BMI, whereas an improved prognosis has been observed in overweight and, more importantly, obese patients [9, 10, 11]. Furthermore, not only pre‐surgical BMI but also pre‐disease BMI were independent prognostic factors, eliminating a possible reverse causation bias [12].

This was first shown in institutional series, but large‐scale analysis of nationwide data sets confirmed this phenomenon [9, 10, 11, 12, 13]. This behaviour has been considered typical of lung cancer and dubbed the ‘lung cancer paradox’ [11, 13].

Analysis of Epithor, the prospective database of the French Society of Thoracic and Cardiovascular Surgery (FSTCVS), showed that the prognostic impact of BMI was independent of possible confounders, including age, sex, WHO performance status, Charlson comorbidity index, tumour side, extent of resection and histological type, as well as stage of disease [13].

BMI is calculated as weight/square of height; in other words, mathematically, BMI and height are inversely related. However, the prognostic significance of height does not reflect this mathematical relationship. In a cohort of 54 631 patients undergoing curative lung resection for lung cancer, we showed that sex‐specific height (sH) quartiles were associated with survival, with taller people having better outcomes [14]. The hypothesised mechanism was that taller stature could be a marker of optimal childhood nutrition with a beneficial effect on the adult immune system. However, it was underlined that other unaccounted‐for confounding factors could explain the observed impact of height on lung cancer outcomes [14]. Adult height significantly affects several social and economic inequalities that could, in turn, influence lung cancer outcomes [14, 15]. In that study on height, we found that sH was independent not only of common confounders but also of BMI. Furthermore, when the population was stratified by BMI categories, the prognostic significance of sH categories was maintained [14]. This led us to question the prognostic value of weight, the other parameter used to calculate BMI, in patients with lung cancer undergoing surgery.

Studying the effect of crude weight on outcome would not be reliable because at the individual level, weight is largely dependent on height and does not reliably reflect obesity or adiposity: This is why forms of ‘relative weight’ (including BMI) are used. For our purposes, we attempted to use weight‐for‐height ratios, which are another way of assessing relative weight: An individual's weight is compared with a reference weight expected for their height, as is commonly used for children and adolescents [16]. However, to our knowledge, this approach has not been proposed to study the effect of weight on lung cancer outcome. The difficulty in using this approach could be represented by the availability of a sufficiently large dataset in an adult population: Practically, it should contain, for each centimetre of height, a sufficient number of patients to allow the calculation of an average weight for a given height and, consequently, the ratio between the observed weight and the average for a given height, and this in each sex. This ratio would thus indicate what the somatic profile of an individual is for each centimetre of height, with ratios < 1 indicating thinness and a ratio ≥ 1 indicating fatness. Previous experience led us to believe that Epithor would meet these criteria, and we undertook the present study to assess the prognostic significance of height, sex‐specific height‐normalised weight (sHNW) and both in resectable lung cancer. We also wanted to compare the prognostic significance of these parameters with BMI.

## 2 Materials and Methods

The study was approved by the FSTCVS Institutional Review Board (CERC‐SFCTCV‐22 February 2021‐Num02_ImpactIMC‐Cancer). Informed consent was obtained for inclusion in the database, and patients were aware that their data would be used for research purposes.

### 2.1 Epithor, the French National Database for General Thoracic Surgery

Created in 2002, Epithor is the official database of the FSTCVS and includes most surgical procedures performed in French thoracic surgery departments. To date, 111 thoracic surgery centres contribute to the updating of the database. The list of centres participating in Epithor in the time frame of the study can be found in the Supplementary Appendix. Data quality monitoring is financially supported by the French National Cancer Institute (INCA). Epithor is endorsed by the French National High Authority for Health (HAS), the government agency responsible for improving the quality of health care and ensuring that the entire health care system provides state‐of‐the‐art care. Previous reports have focused on the technical features of Epithor [13, 14, 17, 18, 19]. In particular, the use of hierarchical pull‐down menus and the absence of free text fields facilitate the completeness and accuracy of the data. Routine utilities are built into the software to ensure data consistency and to warn of anomalous or inconsistent values. A total of 52 variables could be collected per patient, covering information on patient characteristics, comorbidities, lung function, surgical procedures, cancer staging and post‐operative outcomes [13, 14, 17, 18, 19]. The vital status of the patients is made available by automatised interrogation of the website of the French National Institute for Statistics and the Economy (INSEE) [20]. Epithor includes features that allow participating surgeons to benchmark their activity against the national picture by comparing the local database with the national one for completeness. This comparison is expressed as a quality score ranging from 0% to 100%. Since 2010, the accuracy of data collection has been verified by regular external on‐site audits.

### 2.2 Patient Population

The study included 50 653 patients undergoing surgical treatment for stage I–IIIA lung cancer. To account for the progressive implementation of the Epithor project by different surgical centres in 2002, we extracted data from patients aged 18 years or older who underwent upfront surgery with curative intent between 1 January 2003 and 31 December 2017.

### 2.3 Clinical Variables Retrieved

Retrieved patient‐related variables included age, sex, weight, height, BMI, history of tobacco consumption, WHO performance status (WHO PS), Global Initiative for Chronic Obstructive Lung Disease (GOLD) score and American Society of Anesthesia score (ASA score). Surgery‐related variables included the extent of resection, histological type and pathological staging according to the International Association for the Study of Lung Cancer classification. Staging was reattributed according to the eight edition [21]. The date of surgery, but not the date of initial diagnosis, is available in Epithor. Of note, according to the French National Institute of Cancer (INCA) guidelines, surgery is performed within 30 days of diagnosis.

### 2.4 BMI, Height and Height‐Normalised Weight

BMI was calculated as weight/height^2^ and categorised as underweight (< 18.5 kg/m^2^), normal weight (18.5–24.9 kg/m^2^), overweight (25–29.9 kg/m^2^) and obesity (≥ 30 kg/m^2^) [22]. For each sex, height was categorised into sex‐specific quartiles (sH). sHNW was defined as the ratio of an individual's weight to the mean weight of individuals of the same sex and height, and it was categorised into quartiles. Finally, the sum of the category membership (ranging from 1 to 4 according to quartiles) of sH and sHNW was calculated, and the results were categorised into four groups of sH/sHNW, namely lower (sum = 2), intermediate lower (sum = 3 to 4), intermediate higher (sum = 5 to 6) and higher (sum = 7–8).

### 2.5 Outcome Definition

The primary endpoint was overall survival up to 7 years.

### 2.6 Statistical Analysis

Descriptive data were expressed as frequencies and percentages for qualitative variables and as means and standard deviations for continuous variables. Associations between height categories and clinical variables were assessed using ANOVA, Kruskal–Wallis, and *χ* ^2^ tests, and survival curves were constructed using the Kaplan–Meier estimator. Cox proportional hazards regression analysis (only factors significantly associated with survival at univariate analysis were entered) was used to estimate the independent prognostic impact of different variables. Four multivariate models were constructed, each including, together with age, sex, WHO PS, ASA score, extent of resection, histological type, stage of disease, either BMI or height (categorised into four sex‐specific quantiles (sH)) or sHNW or sH/sHNW. GOLD was not introduced because of lack of significance at univariate analysis.

## 3 Results

The study included 50 653 patients. Mean age was 65.61 ± 9.45, and 31.99% were women. WHO PS were 0, 1 and > 1 in 52.59%, 39.66% and 7.75% of patients, respectively. The most frequent histological types were represented by adenocarcinoma and squamous‐cell carcinoma, accounting for 67.76% and 27.25% of cases, respectively. Stage I, II and IIIA diseases accounted for 60.86%, 20.89% and 18.25% of patients. Interventions included lobectomy in 38 464 (75.94%) cases, bilobectomy in 1799 (3.55%), segmentectomy in 4602 (9.09%), pneumonectomy in 3711 (7.33%) and wedge resections in 1916 (3.78%) (Table 1). Table 1 describes main characteristics separately in men and women.

**TABLE 1:** Baseline characteristics of the whole population and with respect to sex.

Features	Total population	Women	Men
	*n* = 50 653	*n* = 16 204 (31.99%)	*n* = 34 449 (68.01%)
Age. mean (SD) years	65.61 (9.45)	64.53 (10.11)	66.12 (9.09)
Tobacco. no. (%)			
Never	4141 (8.18%)	2716 (16.76%)	1425 (4.14%)
Current or former	28 649 (56.56%)	8915 (55.02%)	19 734 (57.28%)
Unknown status	17 863 (35.27%)	4573 (28.22%)	13 290 (38.58%)
Height. mean (SD) cm	169.33 (8.49)	161.47 (6.39)	173.02 (6.63)
Weight. mean (SD) kg	73.19 (15.56)	63.79 (13.69)	77.62 (14.37)
BMI. no. (%)			
Underweight	2229 (4.40%)	1276 (7.88%)	953 (2.77%)
Normal weight	23 265 (45.94%)	8613 (53.16%)	14 652 (42.55%)
Overweight	17 472 (34.50%)	4155 (25.64%)	13 317 (38.67%)
Obesity	7672 (15.15%)	2158 (13.32%)	5514 (16.01%)
WHO PS. no. (%)			
0	25 571 (52.59%)	9511 (60.58%)	16 060 (48.78%)
1	19 286 (39.66%)	5310 (33.82%)	13 976 (42.45%)
2	3451 (7.10%)	810 (5.16%)	2641 (8.02%)
3	307 (0.63%)	68 (0.43%)	239 (0.73%)
4	8 (0.02%)	0 (0.00%)	8 (0.02%)
GOLD. no. (%)			
0	46 720 (92.24%)	14 905 (91.98%)	31 815 (92.35%)
1	1638 (3.23%)	609 (3.76%)	1029 (2.99%)
2	2095 (4.14%)	644 (3.97%)	1451 (4.21%)
3	193 (0.38%)	44 (0.27%)	149 (0.43%)
4	7 (0.01%)	2 (0.01%)	5 (0.01%)
ASA score. no. (%)			
1	7589 (15.07%)	3023 (18.77%)	4566 (13.34%)
2	26 038 (51.72%)	8917 (55.36%)	17 121 (50.01%)
3	16 287 (32.35%)	4072 (25.28%)	12 215 (35.38%)
4	428 (0.85%)	95 (0.59%)	333 (0.97%)
FEV1 (%predicted). mean (SD)	81.13 (21.13)	87.05 (21.46)	78.31 (20.37)
Extent of resection. no. (%)			
Wedge resection	1916 (3.78%)	579 (3.57%)	1337 (3.88%)
Segmentectomy	4602 (9.09%)	1890 (11.66%)	2712 (7.87%)
Lobectomy	38 464 (75.94%)	12 528 (77.31%)	25 936 (75.29%)
Bilobectomy	1799 (3.55%)	466 (2.88%)	1333 (3.87%)
Pneumonectomy	3711 (7.33%)	709 (4.38%)	3002 (8.71%)
Exploration	159 (0.31%)	32 (0.20%)	127 (0.37%)
Histology. no. (%)			
Adenocarcinoma	34 320 (67.76%)	13 352 (82.40%)	20 968 (60.87%)
Squamous cell	13 802 (27.25%)	2174 (13.42%)	11 628 (33.75%)
Large cell	2256 (4.45%)	586 (3.62%)	1670 (4.85%)
Others	275 (0.54%)	92 (0.57%)	183 (0.53%)
Pathological Stage. no. (%)			
IA	19 081 (37.67%)	7117 (43.92%)	11 964 (34.73%)
IB	11 746 (23.19%)	3492 (21.55%)	8254 (123.96%)
II	10 580 (20.89%)	2949 (18.20%)	7631 (22.15%)
IIIA	9246 (18.25%)	2646 (16.33%)	6600 (19.16%)
Score sex‐specific height‐height normalised weight]. no. (%)			
Lower	3486 (6.88%)	1214 (7.49%)	2272 (6.60%)
Lower Intermediate	16 664 (32.90%)	5269 (32.52%)	11 395 (33.08%)
Higher Intermediate	22 118 (43.67%)	6946 (42.87%)	15 172 (44.04%)
Higher	8385 (16.55%)	2775 (17.13%)	5610 (16.28%)

*Note:* Characteristics are reported as number (%) or mean (SD).

Abbreviations: ASA, American Society of Anesthesiologist; BMI, body mass index; FEV1, forced expiratory volume in one second; GOLD, Global Initiative for Obstructive Lung Disease; WHO PS, performance status.

### 3.1 Morphometrics

The mean BMI was 26.32 (SD 4.11); 2229 patients (4.40%) were underweight, 23 265 (45.94%) were normal weight, 17 472 (34.50%) were overweight and 7672 (15.15%) were obese.

The mean height for men and women was 173.02 (SD 6.63) and 161.47 (SD 6.39) cm, respectively. The 25th, 50th (median) and 75th percentiles of height were 169, 173 and 178 cm for men and 157, 161 and 165 cm for women. Table 1 describes main clinical and pathological features and the distribution of classes of BMI and sH/sHNW in the whole population and according to the sex.

Table 2 describes the main characteristics in relation to the four classes of sH/sHNW. Participants in the higher group of sH/sHNW class were younger (64.97 years ± 8.98; *p* < 0.0001). Of note, distribution of BMI categories within sH/sHNW class was significantly different (*p* < 0.0001).

**TABLE 2:** Characteristics of patients with respect to the Score sH/sHNW.

Features	Lower	Lower Intermediate	Higher Intermediate	Higher	p
	*n* = 3486 (6.88%)	n = 16 664 (32.90%)	*n* = 22 118 (43.67%)	*n* = 8385 (16.55%)	
Age. mean (SD) years	65.34 (9.49)	65.76 (9.77)	65.79 (9.37)	64.97 (8.98)	<0.0001
Sex. no. (%)					
Women	1214 (34.83%)	5269 (31.62%)	6946 (31.40%)	2775 (33.09%)	<0.0001
Men	2272 (65.17%)	11 396 (68.38%)	15 172 (68.60%)	5610 (68.01%)	
Tobacco. no. (%)					
Never	232 (6.66%)	1204 (7.23%)	1899 (8.59%)	806 (9.61%)	<0.0001
Current or former	2020 (57.95%)	9438 (56.64%)	12 335 (55.77%)	4856 (57.91%)	
Unknown status	1234 (35.40%)	6022 (36.14%)	7884 (35.365%)	2723 (32.47%)	
Height. mean (SD) cm	160.99 (6.26)	166.08 (7.16)	170.57 (8.02)	175.96 (7.02)	<0.0001
Weight. mean (SD) kg	52.66 (6.82)	63.11 (8.33)	76.84 (11.60)	92.15 (13.36)	<0.0001
BMI. no. (%)					
Underweight	584 (16.77%)	1137 (6.83%)	507 (2.29%)	1 (0.01%)	<0.0001
Normal	2889 (83.23%)	11 682 (70.15%)	8157 (36.88%)	527 (6.29%)	
Overweight	0 (0.00%)	3824 (22.96%)	9132 (41.29%)	4516 (53.86%)	
Obesity	0 (0.00%)	10 (0.06%)	4321 (19.54%)	3341 (39.84%)	
WHO PS. no. (%)					
0	1517 (45.91%)	8147 (51.17%)	11 509 (54.09%)	4398 (54.18%)	<0.0001
1	1437 (43.49%)	6478 (40.69%)	8220 (38.63%)	3151 (38.81%)	
2	321 (9.72%)	1180 (7.41%)	1424 (6.69%)	526 (6.48%)	
3	28 (0.85%)	112 (0.70%)	124 (0.58%)	43 (0.53%)	
4	1 (0.03%)	5 (0.03%)	2 (0.01%)	0 (0.00%)	
GOLD. no. (%)					
0	3153 (90.56%)	15 384 (92.32%)	20 442 (92.42%)	7737 (92.27%)	<0.0001
1	148 (4.25%)	557 (3.34%)	693 (3.13%)	240 (2.86%)	
2	165 (4.73%)	641 (3.85%)	906 (4.10%)	383 (4.57%	
3	16 (0.46%)	79 (0.47%)	74 (0.33%)	24 (0.29%)	
4	0 (0.00%)	3 (0.02%)	3 (0.01%)	1 (0.01%)	
ASA score. no. (%)					
1	426 (12.32%)	2488 (15.02%)	3464 (15.76%)	1211 (14.52%)	<0.0001
2	1745 (50.46%)	8747 (52.81%)	11 358 (51.67%)	4188 (50.23%)	
3	1251 (36.18%)	5211 (31.46%)	6953 (31.63%)	2872 (34.44%)	
4	26 (1.04%)	117 (0.71%)	208 (0.95%)	67 (0.80%)	
FEV1 (%predicted). mean (SD)	80.02 (22.72)	81.34 (21.29)	81.40 (20.80)	80.49 (19.66)	<0.0001
Extent of resection. no. (%)					
Wedge resection	157 (4.51%)	664 (3.98%)	828 (3.74%)	267 (3.18%)	<0.0001
Segmentectomy	288 (8.26%)	1412 (8.47%)	2044 (9.24%)	858 (10.23%)	
Lobectomy	2568 (73.69%)	12 633 (75.81%)	16 817 (76.03%)	6446 (76.88)	
Bilobectomy	148 (4.25%)	595 (3.57%)	794 (3.59%)	262 (3.13%)	
Pneumonectomy	308 (8.84%)	1297 (7.78%)	1585 (7.17%)	521 (6.21%)	
Exploration	16 (0.46%)	63 (0.38%)	50 (0.23%)	30 (0.36%)	
Histology. no. (%)					
Adenocarcinoma	2270 (65.12%)	11.029 (66.18%)	15 180 (68.63%)	5841 (69.66%)	<0.0001
Squamous cell	1055 (30.26)	4726 (28.36%)	5854 (26.47%)	2167 (25.84%)	
Large cell	149 (4.27%)	807 (4.84%)	969 (4.38%)	331 (3.95%)	
Others	12 (0.34%)	102 (0.61%)	115 (0.52%)	46 (0.55%)	
Pathological Stage. no. (%)					
IA	1259 (36.12%)	6173 (37.04%)	8374 (37.86%)	3275 (39.06%)	<0.0001
IB	822 (23.58%)	3946 (23.68%)	5094 (23.03%)	1884 (22.47%)	
II	756 (21.69%)	3493 (20.96%)	4673 (21.13%)	1658 (19.77%)	
IIIA	649 (18.62%)	3052 (18.31%)	3977 (17.98%)	1568 (18.70%)	

*Note:* Characteristics are reported as number (%) or mean (SD).

Abbreviations: ASA, American Society of Anesthesiologist; BMI, body mass index; FEV1, forced expiratory volume in 1 s; GOLD, Global Initiative for Obstructive Lung Disease; WHO PS, performance status.

### 3.2 Survival

In the whole population, 3‐year and 5‐year survival rates were 76.22% (75.83–76.61) and 65.89% (65.42–66.36), respectively. Overall survival in relation to main clinical and pathologic variables is shown in Table 3.

**TABLE 3:** Survival according to main characteristics.

Features	*p*‐value	Crude HR (95% CI)	5‐year overall survival (95% CI)
Age	<0.0001		
≤ 70 years		1 (ref)	68.64% (68.09–69.19)
> 70 years		1.46 (1.43–1.51)	59.60% (58.73–60.48)
Sex	<0.0001		
Women		1 (ref)	79.82% (79.10–80.53)
Men		2.52 (2.43–2.61)	59.78% (59.21–60.35)
Tobacco*	<0.0001		
Never		1 (ref)	76.66% (74.74–78.58)
Current or former		1.60 (1.48–1.72)	67.90% (67.24–68.58)
WHO PS	<0.0001		
0		1 (ref)	72.97% (72.33–73.62)
1		1.56 (1.52–1.61)	61.41% (60.67–62.16)
2		2.09 (2.00–2.20)	50.85% (49.06–52.64)
3		2.99 (2.60–3.43)	38.48% (32.37–44.57)
4		10.00 (5.00–20.00)	0%
GOLD	0.2332		
0–1		1 (ref)	65.87% (65.40–66.34)
2–4		1.05 (0.97–1.13)	64.81% (62.05–67.58)
ASA score	<0.0001		
1		1 (ref)	75.52% (74.46–76.58)
2		1.44 (1.38–1.50)	68.42% (67.79–69.05)
3		2.11 (2.02–2.21)	57.20% (56.30–58.10)
4		2.83 (2.47–3.24)	46.18% (46.13–46.23)
Extent of resection	<0.0001		
Lobectomy		1 (ref)	67.90% (67.37–68.42)
Bilobectomy		1.34 (1.26–1.43)	57.54% (55.13–59.96)
Pneumonectomy		1.66 (1.59–1.73)	48.96% (47.30–50.62)
Wedge resection		1.37 (1.28–1.46)	57.50% (55.06–59.93)
Segmentectomy		0.79 (1.74–0.84)	72.19% (70.36–74.02)
Exploration		2.77 (2.32–3.31)	25.41% (18.41–32.41)
Histology	<0.0001		
Adenocarcinoma		1 (ref)	69.90% (69.34–70.46)
Squamous cell		1.58 (1.54–1.63)	57.85% (56.96–58.73)
Large cell		1.41 (1.33–1.49)	58.01% (52.60–57.00)
Others		1.61 (1.31–1.96)	58.40% (50.92–65.88)
Pathological stage	<0.0001		
IA		1 (ref)	77.07% (76.36–77.78)
IB		1.39 (1.35–1.45)	67.65% (66.73–68.57)
II		1.71 (1.65–1.77)	59.90% (58.86–60.94)
IIIA		2.22 (2.14–2.30)	48.90% (47.79–50.01)
BMI	<0.0001		
Underweight		1.12 (1.05–1.19)	61.18% (58.93–63.42)
Normal weight		1 (ref)	65.09% (64.41–65.78)
Overweight		0.99 (0.97–1.03)	66.80% (66.01–67.59)
Obesity		0.99 (0.95–1.03)	67.52% (66.32–68.72)
Sex‐specific height quartiles	<0.0001		
I		1 (ref)	63.10% (62.19–64.01)
II		0.94 (0.91–0.98)	65.27% (64.39–66.15)
III		0.89 (0.86–0.92)	66.53% (65.61–67.45)
IV		0.77 (0.74–0.81)	69.57% (68.56–70.59)
Sex‐specific height/normalised weight quartiles	<0.0001		
I		1 (ref)	61.38% (60.43–62.32)
II		0.88 (0.85–0.92)	65.57% (64.65–66.49)
III		0.82 (0.79–0.85)	68.41% (67.49–69.32)
IV		0.85 (0.81–0.88)	68.14% (67.21–69.07)
Score sex‐specific height‐height normalised weight	<0.0001		
Lower		1 (ref)	58.65% (56.89–60.45)
Lower Intermediate		0.88 (0.84–0.93)	62.96% (62.15–63.78)
Higher Intermediate		0.76 (0.73–0.80)	67.71% (67.02–68.41)
Higher		0.70 (0.66–0.74)	70.12% (68.98–71.26)

Abbreviations: ASA, American Society of Anesthesiologist; BMI, body mass index; GOLD, Global Initiative for Obstructive Lung Disease; WHO PS, performance status. *Information on tobacco consumption was missing for 17 863 (35.27%) patients.

**FIGURE 1:**
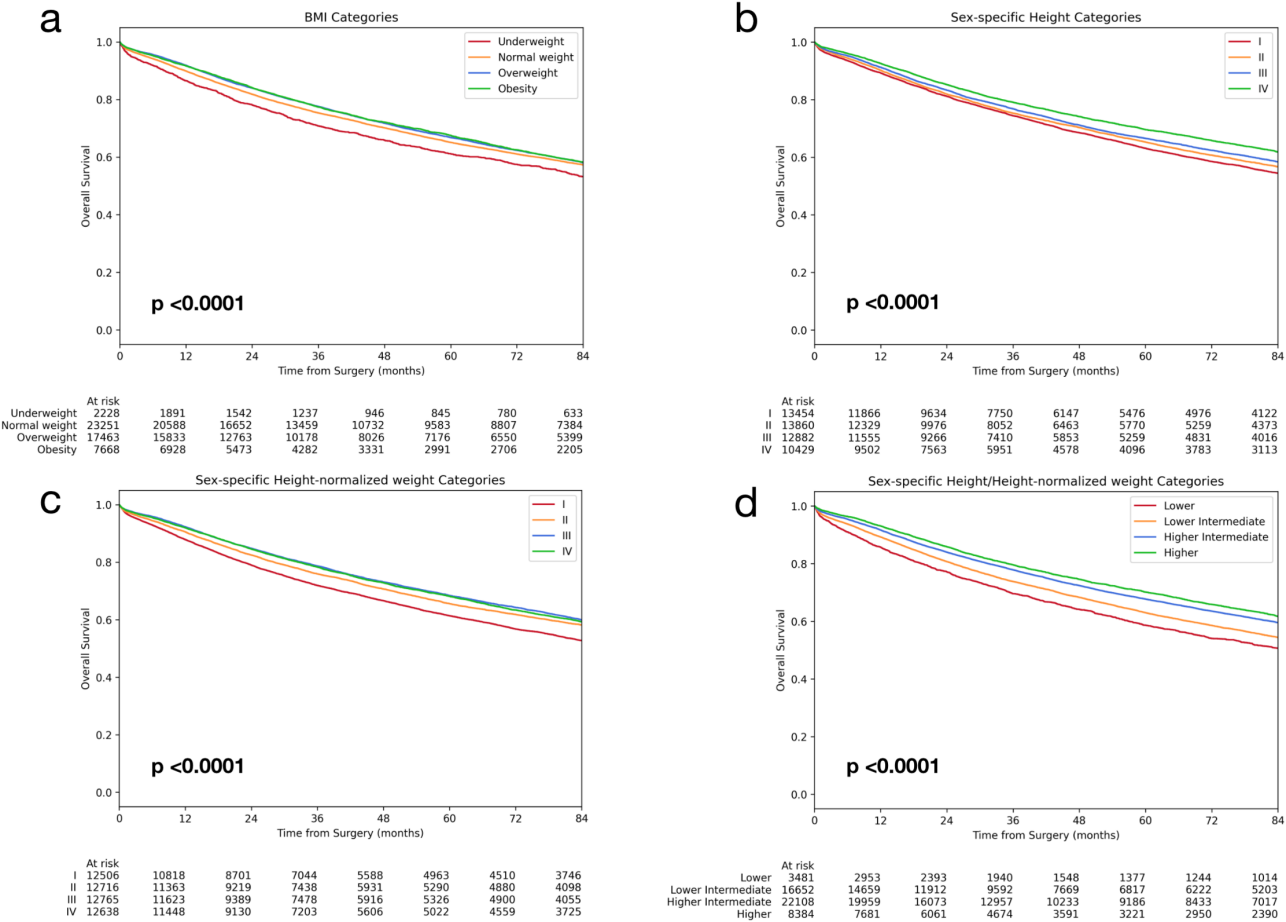
Overall survival of the whole population with respect to body mass index categories (a), sex‐specific height quartiles (sH) (b), sex‐specific height‐normalised weight (sHNW) quartiles (c) and scores of SH/sHNW (d).

BMI was associated with survival, underweight being associated with worse outcome as compared with normal weight and overweight and, at a greater extent, obesity being associated with improved survival (Table 3; Figure 1a). Sex‐specific height (sH) categories also predicted survival, taller height being protective (crude HRs of second, third and fourth quartiles vs. quartiles: 0.94 (95% CI 0.91–0.98), 0.89 (95% CI 0.86–0.92), 0.77 (95% CI 0.74–0.81); *p* < 0.0001) (Figure 1b).

sHNW was strongly associated with long‐term survival, with lower sHNW category being associated with increased risk and higher sHNW categories being protective (Crude HRs of second, third and fourth quartiles vs. first quartile: 0.88 (95% CI 0.85–0.92), 0.82 (95% CI 0.79–0.85), 0.85 (95% CI 0.81–0.88); *p* < 0.0001) (Table 3; Figure 1c).

The four classes of sH/sHNW showed even higher differences in prognosis with respective crude HRs of 0.88 (0.84–0.93), 0.76 (0.73–0.80) and 0.70 (0.66–0.74) in the intermediate lower, intermediate higher and higher classes as compared with the lower class (Table 2; Figure 1d). Five‐year overall survival rates were 58.65% (56.89–60.45), 62.96% (62.15–63.78), 67.71% (67.02–68.41) and 70.12% (68.98–71.26) in the lower, intermediate lower, intermediate higher and higher classes, respectively.

For comparison, crude HRs for BMI category were 1.12 (1.05–1.19), 0.99 (0.97–1.03) and 0.99 (0.95–1.03) (*p* < 0.0001; Table 3) in underweight, overweight and obesity versus normal weight. Five‐year overall survival rates were 61.18% (58.93–63.42), 65.09% (64.41–65.78), 66.80% (66.01–67.59) and 69.57% (68.56–70.59) in underweight, normal weight, overweight and obesity, respectively.

### 3.3 Stratifications

**FIGURE 2:**
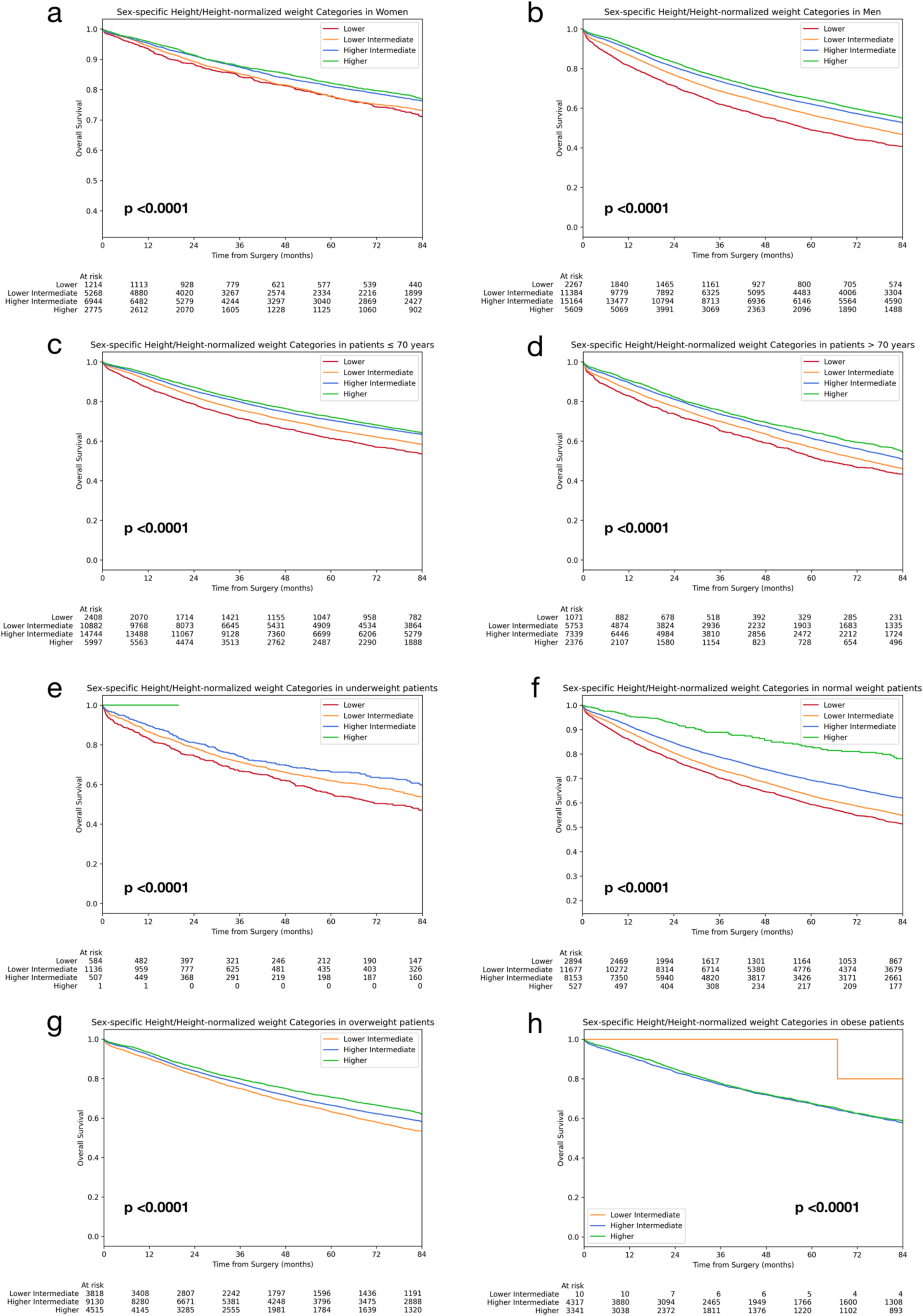
Stratifications to assess the prognostic impact of the score SH/sHNW. OS with respect to SH/sHNW in women (a), men (b), patients aged ≤ 70 years (c), >70 years (d), underweight (e), normal weight (f), overweight (Panel g) and obese (h).

The prognostic impact of sH/sHNW categorisation was further confirmed by stratifying by main clinical and pathological characteristics, including sex (Figure 2a,b) and age (Figure 2c,d) Interestingly, when stratifying for BMI classes, the prognostic discrimination of sH/sHNW categorisation was impressive for underweight (Figure 2e), normal‐weight (Figure 2f) and overweight (Figure 2g).

**FIGURE 3:**
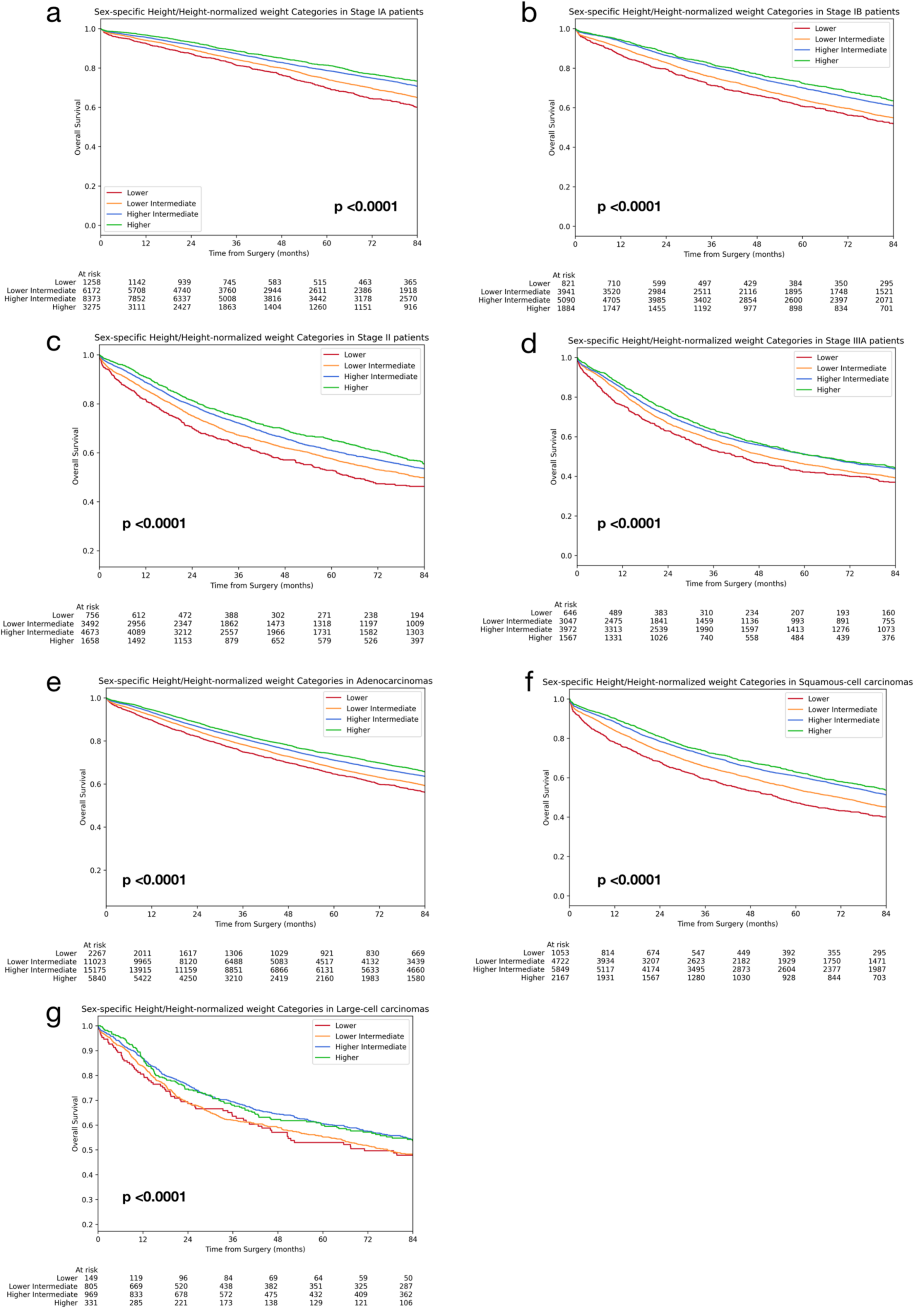
Stratifications to assess the prognostic impact of the score SH/sHNW. OS with respect to SH/sHNW in patients with pathologic stage IA (a), IB (b), II (c), IIIA (d), adenocarcinomas (e), squamous cell carcinomas (f) and large cell carcinomas (g).

Finally, stratification by stage and histology showed that prognostic signification of sH/sHNW categorisation was preserved among stages and principal histologic types (Figure 3).

### 3.4 Multivariable Analysis

Four multivariable models were built. In each model built, we entered age (≤ or > 70 years), sex, WHO PS, ASA score, extent of resection, histology, pathologic stage and either sH (Model 1) or sHNW (Model 2) or BMI (Model 3) or sH/sHNW class (Model 4). The models showed the independent prognostic significance of height, sHNW, sH/HNW score and BMI. sH/s/HNW seemed an excellent independent predictor (Tables 4 and 5).

**TABLE 4:** Cox multivariable analysis. Both models include age, sex, WHO PS, ASA class, extent of resection, histology, and pathologic stage, together with either sex‐specific height quartiles (Model 1) or sex‐specific height‐normalised weight quartiles (Model 2).

	Model 1		Model 2	
Features	*p*‐value	HRs (95% CI)	*p*‐value	HRs (95% CI)
Age				
≤ 70 years		1 (ref)		1 (ref)
> 70 years	< 0.0001	1.38 (1.34–1.42)	< 0.0001	1.42 (1.38–1.47)
Sex				
Women		1 (ref)		1 (ref)
Men	< 0.0001	2.27 (2.19–2.36)	< 0.0001	2.29 (2.20–2.37)
WHO PS				
0		1 (ref)		1 (ref)
1	< 0.0001	1.30 (1.26–1.34)	< 0.0001	1.30 (1.26–1.34)
2	< 0.0001	1.52 (1.44–1.60)	< 0.0001	1.51 (1.43–1.59)
3	< 0.0001	2.00 (1.74–2.31)	< 0.0001	1.94 (1.68–2.24)
4	< 0.0001	6.03 (3.02–12.09)	< 0.0001	5.54 (2.77–11.10)
ASA score				
1		1 (ref)		1 (ref)
2	< 0.0001	1.17 (1.12–1.22)	< 0.0001	1.18 (1.13–1.24)
3	< 0.0001	1.46 (1.39–1.54)	< 0.0001	1.48 (1.41–1.56)
4	< 0.0001	1.69 (1.46–1.95)	< 0.0001	1.74 (1.50–2.01)
Extent of resection				
Lobectomy		1 (ref)		1 (ref)
Bilobectomy	0.1699	1.05 (0.98–1.12)	0.1808	1.05 (0.98–1.12)
Pneumonectomy	< 0.0001	1.17 (1.12–1.23)	< 0.0001	1.17 (1.11–1.22)
Wedge resection	< 0.0001	1.39 (1.30–1.49)	< 0.0001	1.39 (1.30–1.49)
Segmentectomy	0.0889	0.95 (0.89–1.01)	0.0945	0.95 (0.89–1.01)
Exploration	< 0.0001	1.64 (1.36–1.98)	< 0.0001	1.66 (1.34–2.00)
Histology				
Adenocarcinoma		1 (ref)		1 (ref)
Squamous cell	< 0.0001	1.13 (1.10–1.17)	< 0.0001	1.14 (1.10–1.18)
Large cell	< 0.0001	1.18 (1.11–1.26)	< 0.0001	1.17 (1.10–1.24)
Others	0.0003	1.45 (1.19–1.78)	0.0002	1.47 (1.20–1.79)
Pathological stage				
IA		1 (ref)		1 (ref)
IB	< 0.0001	1.30 (1.25–1.35)	< 0.0001	1.30 (1.25–1.35)
II	< 0.0001	1.53 (1.47–1.60)	< 0.0001	1.52 (1.46–1.58)
IIIA	< 0.0001	2.05 (1.96–2.13)	< 0.0001	2.05 (1.97–2.13)
Sex‐specific height quartiles				
I		1 (ref)	—	—
II	0.0966	0.97 (0.93–1.01)	—	—
III	0.0310	0.96 (0.92–1.00)	—	—
IV	< 0.0001	0.88 (0.85–0.92)	—	—
II		1 (ref)	—	—
III	0.5761	0.99 (0.95–1.03)	—	—
IV	< 0.0001	0.91 (0.87–0.95)	—	—
III		1 (ref)	—	—
IV	0.0002	1.08 (1.04–1.13)	—	—
Sex‐specific height/normalised weight quartiles				
I	—	—		1 (ref)
II	—	—	< 0.0001	0.87 (0.84–0.90)
III	—	—	< 0.0001	0.78 (0.75–0.81)
IV	—	—	< 0.0001	0.77 (0.74–0.80)
II	—	—		1 (ref)
III	—	—	< 0.0001	0.90 (0.87–0.94)
IV	—	—	< 0.0001	0.89 (0.85–0.92)
III	—	—		1 (ref)
IV	—	—	0.4020	0.98 (0.94–1.02)

Abbreviations: ASA, American Society of Anesthesiologist; BMI, body mass index; GOLD, Global Initiative for Obstructive Lung Disease; WHO PS, performance status.

**TABLE 5:** Cox multivariable analysis. Both models include age, sex, WHO PS, ASA class, extent of resection, histology, and pathologic stage, together with either BMI (Model 3) or the score of sex‐specific height‐height normalised weight (Model 4).

	Model 3		Model 4	
Features	*p*‐value	HRs (95% CI)	*P*‐value	HRs (95% CI)
Age				
≤ 70 years		1 (ref)		1 (ref)
> 70 years	< 0.0001	1.43 (1.38–1.47)	< 0.0001	1.39 (1.35–1.43)
Sex				
Women		1 (ref)		1 (ref)
Men	< 0.0001	2.37 (2.28–2.46)	< 0.0001	2.29 (2.20–2.37)
WHO PS				
0		1 (ref)		1 (ref)
1	< 0.0001	1.30 (1.26–1.34)	< 0.0001	1.30 (1.26–1.34)
2	< 0.0001	1.51 (1.44–1.59)	< 0.0001	1.50 (1.43–1.58)
3	< 0.0001	1.95 (1.69–2.26)	< 0.0001	1.98 (1.72–2.29)
4	0.3863	5.48 (2.74–10.98)	< 0.0001	5.56 (2.78–11.14)
ASA score				
1	< 0.0001	1 (ref)		1 (ref)
2	< 0.0001	1.18 (1.13–1.23)	< 0.0001	1.17 (1.12–1.23)
3	< 0.0001	1.48 (1.41–1.55)	< 0.0001	1.47 (1.40–1.55)
4	< 0.0001	1.72 (1.48–1.99)	< 0.0001	1.72 (1.49–1.99)
Extent of resection				
Lobectomy		1 (ref)		1 (ref)
Bilobectomy	0.2027	1.04 (0.98–1.12)	0.1744	1.05 (0.98–1.12)
Pneumonectomy	< 0.0001	1.17 (1.11–1.23)	< 0.0001	1.19 (1.14–1.24)
Segmentectomy	0.0852	0.95 (0.89–1.01)	0.0979	0.95 (0.89–1.01)
Wedge resection	< 0.0001	1.39 (1.30–1.49)	< 0.0001	1.39 (1.30–1.49)
Exploration	< 0.0001	1.68 (1.39–2.02)	< 0.0001	1.62 (1.34–1.95)
Histology				
Adenocarcinoma		1 (ref)		1 (ref)
Squamous cell	< 0.0001	1.14 (1.11–1.18)	< 0.0001	1.13 (1.01–1.17)
Large cell	< 0.0001	1.17 (1.10–1.24)	< 0.0001	1.17 (1.10–1.25)
Others	0.0003	1.46 (1.19–1.78)	0.0003	1.45 (1.19–1.78)
Pathological stage				
IA		1 (ref)		1 (ref)
IB	< 0.0001	1.30 (1.25–1.35)	< 0.0001	1.30 (1.25–1.35)
II	< 0.0001	1.52 (1.46–1.58)	< 0.0001	1.53 (1.47–1.59)
IIIA	< 0.0001	2.05 (1.97–2.13)	< 0.0001	2.05 (1.97–2.14)
BMI				
Underweight	< 0.0001	1.33 (1.24–1.43)	—	—
Normal weight		1 (ref)	—	—
Overweight	< 0.0001	0.87 (0.84–0.89)	—	—
Obesity	< 0.0001	0.84 (0.80–0.87)	—	—
Underweight		1 (ref)	—	—
Overweight	< 0.0001	0.65 (0.61–0.70)	—	—
Obesity	< 0.0001	0.63 (0.58–0.68)	—	—
Overweight		1 (ref)	—	—
Obesity	0.1255	0.97 (0.93–1.01)	—	—
Score sex‐specific height‐height normalised weight				
Lower	—	—		1 (ref)
Lower Intermediate	—	—	< 0.0001	0.88 (0.83–0.93)
Higher Intermediate	—	—	< 0.0001	0.76 (0.72–0.81)
Higher	—	—	< 0.0001	0.71 (0.67–0.76)
Lower Intermediate	—	—		1 (ref)
Higher Intermediate	—	—	< 0.0001	0.87 (0.84–0.90)
Higher	—	—	< 0.0001	0.81 (0.78–0.85)
Higher Intermediate	—	—		1 (ref)
Higher	—	—	0.0016	0.93 (0.89–0.97)

Abbreviations: ASA, American Society of Anesthesiologist; BMI, body mass index; GOLD, Global Initiative for Obstructive Lung Disease; WHO PS, performance status.

## 4 Discussion

In this study, we report evidence that sHNW is an independent predictor of survival in patients undergoing surgery for NSCLC. We confirmed in this dataset that height was also an independent prognostic factor and showed that taking into account both sex‐specific height (sH) categories and sHNW allowed identification of a third parameter (sH/sHNW) whose discriminatory power in terms of overall survival was remarkably higher than the latter. Very interestingly, we showed that among patients with normal BMI, the sH/sHNW scoring allowed identifying subgroups with impressive prognostic differences. This strongly supports the concepts that patient‐related factors are as important as tumour‐specific factors in determining outcome and that simple anthropometric measures could play a useful role in both epidemiology and clinical practice. This could represent a significant complement to information provided by BMI calculation.

BMI can be considered a marker of somatic profile more than of mere adiposity, because of the impossibility (as weight) of differentiating the fat from the lean mass. BMI can provide the idea of somatic profile at an individual level, whereas weight cannot reliably, because weight strictly especially relays on height in everyone. We previously reported that increasing height is an independent predictor of better survival, although BMI is calculated as weight/square height [14], and we confirm the prognostic value of height in this dataset. Although height is a highly heritable, classic polygenic trait with approximately 700 common associated variants identified through genome‐wide association studies [23], adult height is also considered a useful proxy measure of childhood nutrition and disease burden. Taller stature has often been associated with decreased risk of all‐cause mortality [24], but with an overall increased risk of cancer development [25, 26, 27, 28]: thus, our finding of prognostic significance of height prompted us to suggest that this could represent a novel aspect of the lung cancer paradox [14]. We showed herein that in resectable lung cancer, both sH and sHNW independently predicted survival and taking into account both (by the sH/sHNW parameter) obviously resulted in a stronger prognostic discrimination.

Mechanisms responsible for the protective effects of higher height and weight on lung cancer remain speculative: It has been shown that, in patients with resectable lung cancer, BMI is inversely correlated with plasma C‐reactive protein levels [9] and directly with prealbumin levels [5] as well as with total psoas or total muscular areas [9, 29], suggesting that lung cancer patients with higher BMI have a more preserved nutritional status and less frequent systemic inflammation and sarcopenia, these parameters representing positive and negative predictive factors, respectively [6, 7, 8, 10]. On the other hand, as previously suggested [14], it should be emphasised that height, although easy to measure, is a complex phenotype that is downstream of multiple biological and sociological determinants: Despite a strong genetic component, the environment, especially economic development and nutrition, plays a major role at the population level in determining adult height [23]. If we can hypothesise that greater height could be a marker of good childhood nutrition, with a positive impact on adult immune system function [14], we cannot exclude that other, unaccounted for confounding factors could explain the impact of height on lung cancer outcome: Adult height has an important impact on several social and economic inequalities, which in turn could influence lung cancer outcome [15]. In agreement with this hypothesis, we underline that in a review paper, Perkins et al. showed that evidence across studies indicates that short adult height is driven by environmental conditions, especially net nutrition during early years. That review suggested that adult height is a useful marker of variation in cumulative net nutrition, biological deprivation and standard of living between and within populations [30].

Our study was based on an analysis of a recent, prospectively collected, nationwide database developed mainly for clinical purposes. The men/women ratio of our population reflects current epidemiology in France [31]. Epithor database allowed the collection of potentially confounding clinical and pathological variables that are generally not collected in epidemiological databases. We also showed that the prognostic significance of sH, sHNW and sH/sHNW was independent of the ‘classical’ prognostic factors of resectable lung cancer, including pathological stage, histological type, extent of resection, sex, age, performance status or ASA score, while the prognostic value of all these factors was confirmed. Furthermore, the prognostic impact of sH, sHNW and sH/sHNW on outcome remained evident when stratifying for the above factors. Thus, this phenomenon could represent a novel representation of the ‘lung cancer paradox’.

Main strength of our study is the high number of enrolled patients in a nationwide setting inside a multicentric programme of wide clinical and pathological data collection. Few data were missing with respect to almost all parameters taken into account in our study, but information on exact smoking status was missing in a significant percentage of cases. Similarly, information on PDL1 status and presence of driver oncogenic activation was frequently lacking and anyway not performed at the beginning of our study period. However, we think that this does not affect reliability of our results. The main limitation is the absence of information regarding causes of death, precluding assessment of the impact of sH, sHNW, and sH/sHNW on cancer‐related mortality. Our study has two other limitations: Firstly, adjuvant treatments are not recorded in the Epithor database because these treatments are performed under the care of referring pneumologists, often in centres outside hospitals with thoracic surgery facilities. The second limitation is the lack of information on cancer recurrence; only overall survival is assessed. In any case, outside clinical trials, information on the time of recurrence is often unreliable because of differences in follow‐up strategies (timing and imaging) between centres, which may lead to a significant delay in some centres and an early diagnosis of recurrence in others. However, in an unselected lung cancer population, long‐term deaths are mainly related to cancer‐specific events. [32]. Regardless of this consideration we think that on epidemiological grounds, pending validation in other countries, sH, sHNW, and sH/sHNW should be considered prognostic factors of resectable NSCLC to be taken into account. For further clinical development, the availability of information on mean weight for each centimetre of height in a given population would allow physicians to use sHNW and sH/sHNW, which could be useful and reliable parameters to add to BMI in the basic armamentarium of morphometric assessment.

## Ethics Statement

The Institutional Review Board of the FSTCVS approved the study (CERC‐SFCTCV‐22 February 2021‐Num02_ImpactIMC‐Cancer).

## Conflicts of Interests

The authors declare no conflicts of interest.

## Supporting information


**Data S1:** Supplementary Information.

## Data Availability

Data are available after formal request to and acceptance by the French Society of Thoracic and Cardiovascular Surgery.
