# Supplementary material for: Beyond Body Mass Index: The Impact of Height and Height‐Normalised Weight on Overall Survival of Lung Cancer Undergoing Surgery
Source: J Cachexia Sarcopenia Muscle. 2025 Aug 18;16(4):e70049. doi: 10.1002/jcsm.70049 (PMC12360959; doi:10.1002/jcsm.70049)
Supplement: Supplementary file 1 — Data S1: Supplementary Information. [file JCSM-16-e70049-s001.docx]

List of 2023 Contributor Centers to EPITHOR

| **Contact surgeon** | **Institution** |
| --- | --- |
|  |  |
| Alessio Mariuolo | Institut Mutualiste Mountsouris-Paris |
| Ahed Fallouh | Hôpital René Dubos - Pontoise |
| Albéric de Lambert | Médipôle de Savoie – Challes-les-Eaux |
| Ana Claudia Vlas | CHR d’Orléans |
| Antoine Claret | Hôpital Saint Philibert - Lille |
| Antoine Defontaine | Clinique Mutualiste de Bretagne Occidentale - Quimper |
| Antoine Legras | CHU-Hopital Trousseau - Tours |
| Antonio Minniti | Centre Hospitalier François Mitterrand - Pau |
| Arnaud Pforr | Hôpital Henri Duffaut - Avignon |
| Aurelien Moret | CHG - Chambery |
| Axel Aubert | Institut Alpin du Thorax - Saint-Martin-d'Hères |
| Bastien Orsini | Centre Hospitalier Privé Clairval- Marseille |
| Benoît Delepine | CHU-Hospital Robert Debré- Reims |
| Benoît Lahon | Clinique Belharra - Bayonne |
| Bertrand Richard De Latour | CHU/Polyclinique Saint-Laurent- Rennes |
| Bogdan Berbescu | CHG- Mulhouse |
| Boriana Pektova-Marteau | CHG - Toulon |
| Catalin Cosma | Centre Hospitalier Universitaire Caremeau- Nimes |
| Cedric Perrotin | Clinique Saint-George- Nice |
| Christophe Berton | Pole Santé Oréliance- Orléans |
| Christophe Jayle | CHU –Site De La Millétrie - Poitiers |
| Christophe Klein | Polyclinique Bordeaux Nord-Aquitaine- Bordeaux |
| Christophe Lancelin | Clinique Du Grand Large- Brest |
| Dan Angelescu | CHG de Périgueux |
| David Kaczmarek | Hôpital Privé de la Loire – Saint Etienne |
| Edouard Sage | Hopital Foch- Suresnes |
| Emmanuel Martinod | APHP – Hôpital Avicenne- Bobigny |
| Eric Brechet | Site Emailleurs Colombier- Limoges |
| Eric De La Roche | Clinique De La Sauvegarde- Villeurbanne |
| Eric Mensier | Polyclinique Du Bois- Marcq en Baroeul |
| Florence de Dominicis | CHU Amiens |
| Florence Mazeres | Centre Hospitalier de La Côte Basque - Bayonne |
| Florent Charot | Nouvelle Clinique De L’union- Toulouse |
| Francoise Le Pimpec-Barthes | APHP – Hôpital Europeen Georges Pompidou- Paris |
| Gaetan Singier | Hôpital Privé Jean Mermoz - Lyon |
| Gilles Cardot | Hôpital Duchenne – Boulogne sur Mer |
| Gilles Grosdidier | Polyclinique De Gentilly- Nancy |
| Guillaume Boddaert | Hôpital Percy - Clamart |
| Ilies Bouabdallah | Hôpital Saint Joseph- Marseille |
| Jacques Jougon | CHU – Hôpital du Haut Leveque- Bordeaux |
| Jalal Assouad | APHP – Hôpital Tenon-Paris |
| Jean François Levi | Hôpital Américain – Neuilly sur Seine |
| Jean Marc Baste | CHU – Hopital Charles Nicolle- Rouen |
| Jean Michel Maury | Hôpital Cardio-Vasculaire et Pneumologique Louis Pradel- Bron |
| Jean Philippe Arigon | Clinique Saint Augustin- Nantes |
| Jean-Noel Choplain | CHU- Brest |
| Jean-Philippe Avaro | Hôpital D’instruction Des Armées Sainte- Anne - Toulon |
| Jean-Philippe Berthet | CHU – Hopital Pasteur - Nice |
| Jean-Philippe Le Rochais | CHU Côte de Nacre- Caen |
| Jocelyn Bellier | Hôpital Privé Les Bonnettes - Arras |
| Joel Riviere | Polyclinique du Cotentin - Cherbourg |
| Karel Pfeuty | Centre Hospitalier Yves Le Foll – Saint Brieu |
| Laurence Solovei | CHU – Hôpital Arnaud De Villeneuve- Montpellier |
| Laurent Brouchet | CHU – Hôpital Larrey, Toulouse |
| Lotfi Benhamed | Centre Hospitalier - Valenciennes |
| Lucia Mazzoni | Clinique Saint-Hilaire Esquirol- Agen |
| Luciano Eraldi | Clinique Ambroise Paré - Beuvry |
| Lucile Gust | Hôpital Privé de Provence – Aix en Provence |
| Madalina Grigoroiu | Hôpital Privé - Anthony |
| Maher Dabboussi | Centre hospitalier Jacques Cœur - Bourges |
| Marc Filaire | Centre Jean Perrin- Clermont Ferrand |
| Marco Alifano | APHP-Hôpital Cochin, Paris |
| Marion Durand | Hôpital Ambroise Paré – Neuilly sur Seine |
| Mathieu Coblence | Hôpital Privé Claude Galien - Quincy-sous-Sénart |
| Matthieu Peret | Clinique saint Pierre - Perpignan |
| Mayeul Tabutin | Centre Léon Bérard- Lyon |
| Michel Alauzen | Clinique du Millénaire- Montpellier |
| Myriam Ammi | Centre Hospitalier Universitaire, Angers |
| Nicola Santelmo | Clinique Rhéna - Strasbourg |
| Nicolas Venissac | CHU-Hopital Calmette- Lille |
| Nidal Alsit | Hôpital privé Nancy Lorraine |
| Olaf Mercier | Centre Chirurgical Marie Lannelongue – Le Plessis Robinson |
| Olivier Aze | Centre Hospitalier- Aix en Provence |
| Olivier Hagry | Clinique Sainte Marie – Chalon sur Saône |
| Olivier Pagès | Clinique des Cèdres - Cornebarrieu Grand Toulouse |
| Olivier Tiffet | CHU – Hôpital Nord- Saint-Etienne |
| Pascal Thomas | APHM – Hôpital Nord, Marseille |
| Patrick Bagan | Centre Hospitalier Victor Dupouy- Argenteuil |
| Philippe Boitet | Hopital Privé De L’ Estuaire- Le Havre |
| Philippe Dalous | Clinique d’Occitanie- Muret |
| Philippe Kleinmann | Clinique du Val d’Or – Saint Cloud |
| Philippe Lacoste | Hôpital Nord - Nantes |
| Philippe Rinieri | Clinique Du Cèdre- Bois-Guillaume |
| Pierre Mordant | Groupe Hospitalier Bichat-Claude Bernard- Paris |
| Pierre Tesson | Clinique du Ter - Ploemeur |
| Pierre-Benoît Pages | CHU François Mitterrand- Dijon |
| Pierre-Emmanuel Falcoz | CHU – Nouvel Hôpital Civil - Strasbourg |
| Pierre-Mathieu Bonnet | Hôpital Européen- Marseille |
| Pierre-Yves Brichon | Centre Hospitalier Universitaire -Grenoble |
| Salam Abou Taam | Hôpital Privé Claude Galien Quincy-sous-Sénart |
| Sebastien Franco | CHG- La Rochelle |
| Sophie Guinard | CHG - Colmart |
| Sophie Jaillard-Thery | Hôpital Prive Le Bois- Lille |
| Stéphane Renaud | CHU – Hôpital Brabois, Nancy |
| Valentine Anne | Site Robert Schuman- Metz |
| Vincent Blin | Hôpital Privé Océane- Vannes |
| Vincent Casanova | CHG Bastia |
| Xavier De Kerangal | Hôpital Jacques Monod- Le Havre |
